# Infrared thermography for the analysis of ocular surface temperature after phacoemulsification

**DOI:** 10.5935/0004-2749.20200035

**Published:** 2020

**Authors:** Anna Modrzejewska, Łukasz Cieszyński, Daniel Zaborski, Mirosław Parafiniuk

**Affiliations:** 1 Department of Ophthalmology, Pomeranian Medical University, Szczecin, Poland; 2 Faculty of Computer Science and Information Technology, West Pomeranian University of Technology, Szczecin, Poland; 3 Faculty of Biotechnology and Animal Husbandry, West Pomeranian University of Technology, Szczecin, Poland; 4 Forensic Medicine Institution, Pomeranian Medical University, Szczecin, Poland

**Keywords:** Phacoemulsification, Thermography, Cornea, Body Temperature, Facoemulsificação, Termografia, Córnea, Temperatura corporal

## Abstract

**Purpose:**

In this study, we present our observations on changes in the surface temperature of the cornea, eye, and orbital cavity after cataract surgery.

**Methods:**

A total of 39 patients who underwent cataract surgery based on phacoemulsification were enrolled. Temperature was measured at the center of the cornea, on the eye surface, and in orbital cavities using the FLIR T640 thermal imaging camera at days 1, 14, and 28 after cataract phacoemulsification and compared with preoperative baseline values.

**Results:**

The mean value of ocular surface temperature of the orbital cavity 14 days after cataract surgery was significantly different compared with the preoperative temperature (p≤0.05). Temperature of the investigated areas showed a reduction, with the greatest decrease on day 14 after surgery, followed by an increase on day 28 after surgery, which was comparable to the temperature measured prior to surgery.

**Conclusions:**

The reduction in ocular surface temperature toward the end of post-cataract surgery follow-up may be associated with increased instability of the tear film after phacoemulsification. Therefore, patient awareness regarding the possibility of clinical symptoms of dry eye syndrome during the first month after surgery should be part of clinical management of cataract surgery. Ocular surface temperature did not increase after cataract surgery, suggesting the absence of significant inflammation, and the temperature about 1 month after cataract surgery was comparable to that before surgery. Nevertheless, the negative correlation between age and ocular surface temperature should be of concern in the elderly.

## Abstract (pt)

**Objetivo:**

Este artigo descreve observações sobre mudanças na temperatura da superfície da córnea, olho e cavidade orbital após a cirurgia de catarata.

**Métodos:**

39 pacientes, previamente submetidos à cirurgia de catarata com base em facoemulsificação, foram incluídos no estudo. A temperatura foi medida no centro da córnea, na superfície do olho e nas cavidades orbitárias, com câmera de imagem térmica FLIR T640 nos dias -1, 14 e 28 após a facoemulsificação da catarata e comparada aos valores basais pré-operatórios.

**Resultados:**

Diferenças es tatisticamente significantes foram encontradas apenas para o valor médio da temperatura superficial mediana da cavidade orbital 14 dias após a cirurgia de catarata, em comparação com a temperatura pré-operatória (p≤0,05). A análise revelou uma tendência decrescente na temperatura das áreas investigadas, com a maior diminuição no 14º dia após a cirurgia, seguida por um aumento de temperatura comparável à medida antes da cirurgia no dia 28 após a cirurgia.

**Conclusões:**

A temperatura da superfície ocular após a cirurgia de catarata dimimuiu gradativamente até o final do acompanhamento pós-operatório. A queda de temperatura pode estar associada ao aumento da instabilidade do filme lacrimal. Cerca de um mês após a cirurgia de catarata, a temperatura superficial ocular foi comparável à temperatura medida antes da cirurgia. A temperatura superficial ocular não aumentou após a cirurgia de catarata, sugerindo que não houve aumento significativo na reação inflamatória. Houve uma tendência de correlação negativa entre a idade e a temperatura da superfície ocular. Devido ao aumento da instabilidade do filme lacrimal após a facoemulsificação, deve-se considerar informar o paciente sobre a possibilidade de sintomas clínicos da síndrome do olho seco durante o primeiro mês após a cirurgia.

## INTRODUCTION

Cataract surgery is the most frequently performed ophthalmic micro-invasive surgery worldwide. Coaxial phacoemulsification with simultaneous implantation of a foldable intraocular lens is the most popular technique used for this purpose and the gold standard in modern cataract surgery. A modification of this technique by introducing a microincision (2.6 mm or 2.2 mm) is considered an important achievement in ophthalmic microsurgery. Increased efficacy and safety of this procedure, as well as minimized risk of complications have been achieved through the modern approach to cataract surgery, including the use of innovative surgical techniques, surgical instruments, and intraoperative and perioperative drugs to ensure the best possible functional results in postoperative visual acuity.

Coaxial phacoemulsification results in changes in the surface temperature of the cornea, eye, and orbital cavity. The objective of this study was to measure these changes in ocular surface temperature was measured at 1, 14, and 28 days and compared with preoperative baseline values in individual patients after cataract phacoemulsification at different points of the eye and orbital cavity using with the FLIR T640 thermal imaging camera.

## METHODS

This was a prospective study that included 39 patients who underwent uncomplicated cataract surgery with a standard corneal incision and phacoemulsification using the Bausch & Lomb Stellarisphaco machine (Bausch & Lomb, USA). Subjects with ophthalmological conditions such as dry eye syndrome, glaucoma, ocular inflammation, age-related macular degeneration, endophthalmitis, intraocular hemorrhage or retinal detachment po tentially disturbing preoperative or postoperative ther moemission measured on the ocular surface, were excluded from the study. Patients with systemic infection or fever were also excluded. After excluding patients with incomplete data or extreme values, 36 patients were chosen for analysis isotherms data.

The following tests were done to rule out dry eye syndrome before cataract surgery: Ocular Surface Disease Index (OSDI) (pathological value score >12), Schirmer test (STI) without anesthesia (pathological value <5 mm length wet paper after 5 min of the test), and tear breakup time (TBUT) (pathological value <10 s). Dry eye assessment and diagnosis followed the recommendations and guidelines set up by the International Task Force Delphi Panel and the International Dry Eye Workshop^(1)^. Postoperatively, all patients used the same topical medications (fluoroquinolone, steroidal, and nonsteroidal anti-inflammatory eye drops).

**Figure 1:**
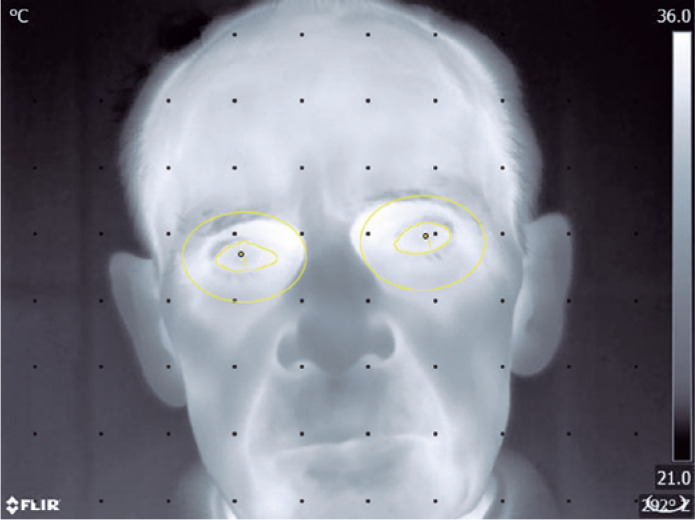
Gray-scale thermographic image of a patient’s face on the ImageJ program.

Measurements were taken in the same patient to eliminate the effect of inter-individual variability that controlled for several potential confounding factors and optimized the reliability of the test. Temperature was measured one day preoperatively and 1, 14, and 28 days after cataract surgery. Eye drops were not used on the day of the test. All patients were acclimatized to the cli nical environment for at least 15 min, and thermographic analysis was done at the same time (8-10 am) to avoid daily temperature variations, in a room with a stable temperature of 22°C (~72°F), and relatively stable air humidity and light intensity. Three temperature measurements were taken at a distance of 1.0 m with a FLIR T640 thermographic camera equipped with a 25-mm lens (FLIR Systems Inc., Boston, MA, USA), 3 s after eye opening, perpendicular to the examined area. The FLIR T640 camera had a 640 × 480-pixel detector, with a maximum frame rate of 30 Hz, thermal sensitivity <30 mK (<0.03°C), spatial resolution of 0.68 mrad, and measurement accuracy of ±2%. The temperature values were read out from the thermograms using the FLIR Tools software (FLIR Systems Inc., Boston, MA, USA). The target emissivity was set to 0.95, a value commonly used for biological tissues (e.g., Martello et al., 2016). Using ImageJ software (National Institutes of Health, Bethesda, MD, USA), the gray-scale images (values ranging from 0 to 255) were calibrated with a straight line function to obtain the temperatures expressed in degree Celsius (by comparing the minimum and maximum temperature values with their corresponding gray-scale values) (Figure 1). The thermograms were subsequently processed in the Matlab environment (R2017a, MathWorks Inc., Natick, MA, USA). The following regions of interest were analyzed: the center of the cornea in the left or right eyes (point), left or right eyes (area) and left or right orbital cavities (area). The area of the eye was delineated manually after the superimposition of the thermographic image on the optical image, as the surface of the eye between eyelids. The last area was delineated as an ellipse with a minor axis equal to double the distance between the center of the pupil and the upper edge of the eye, and the major axis equal to 0.6 of the distance between the center of the pupil and the left/right margin of the eye and superimposed onto the optical image.

The protocol was approved by the Bioethics Committee of the Medical University (Approval No. KB-0012/ 141/15).

### Statistical analysis

For each area, the following statistical parameters of the surface temperature were calculated: mean, standard deviation, minimum, maximum, median, and mode. Results obtained from the thermal camera at individual time points (1, 14, and 28 days after cataract surgery) were compared with the values obtained one day before surgery. A one-way analysis of variance (ANOVA) with repeated measures was used for statistical analysis. The median temperature was selected for further study due to its desired properties (insensitivity to outliers and robust statistically significant correlations with the remaining temperature parameters). To determine diffe rences in the mean values of the surface temperature median between the first time point (one day before cataract surgery) and the remaining time points (1, 14, and 28 days after cataract surgery), Dunnett’s post-hoc test was applied. Correlations between the median surface temperature and patients’ age at individual time points were calculated based on the Pearson correlation coeffi cient, or the Spearman’s rank correlation coefficient for non-normally distributed data.

For each patient, the highest and the lowest isotherms were determined, which surrounded an area with the same temperature in the region limited by the line of the eyebrows and the wings of the nostril. In addition, the areas between the isotherms were estimated (expressed as the number of pixels) and the ratios between them were calculated by dividing the number of pixels of the area surrounded by the higher isotherm by the number of pixels of the area surrounded by the lower isotherm. A one-way ANOVA with repeated measures was applied to analyze differences in the mean ratios between the isothermal areas (originally expressed as the number of pixels). Values were expressed as mean ± SD. All calculations were performed using Statistica software (v. 13, Dell Inc., Tulsa, OK, USA). Statistical significance was adopted at p≤0.05.

## RESULTS

Thirty-nine patients with a temporal 2.6 mm corneal incision during phacoemulsification were included. The mean age of all patients was 72.6 ± 8.18 years; females aged 73.9 ± 8.0 years and males aged 69.7 ± 8.3 years. Data analyzed from 27 (69.2%) females and 12 (30.8%) males showed a mean OSDI of 3.82 ± 1.80, STI without anesthesia of 7.85 ± 2.96 mm, and TBUT of 10.74 ± 1.29 s. The operating time was comparable in all patients. The tests performed to rule out dry eye syndrome before cataract surgery showed normal results for all patients.

Results of the thermographic analysis of the eyes before and after cataract surgery are presented in table 1. Significant differences were found only for the mean value of median surface temperature of the orbital cavity 14 days after cataract surgery as compared with the preoperative temperature (p≤0.05). A decreasing trend in the temperature of the investigated areas was observed, with the greatest decrease on day 14 after surgery, followed by an increase, on day 28 after surgery, to a temperature that was comparable to that measured before surgery. The lowest temperature was measured in the center of the cornea and the highest in the orbital cavity.

**Table 1:** Values of temperature parameters for the three regions of the eye at individual time points pre and post-cataract surgery (n=39)

	Surgery days Variable	Day -1		Day 1		Day 14		Day 28	
		Mean	SD	Mean	SD	Mean	SD	Mean	SD
Center of the cornea	Mean	33.48	1.15	33.39	1.33	33.17	1.06	33.49	1.21
	SD	0.00	0.00	0.00	0.00	0.00	0.00	0.00	0.00
	Min	33.48	1.15	33.39	1.33	33.17	1.06	33.49	1.21
	Max	33.48	1.15	33.39	1.33	33.17	1.06	33.49	1.21
	Median^1^	**33.48**	**1.15**	**33.39**	**1.33**	**33.17**	**1.06**	**33.49**	**1.21**
	Mode	33.48	1.15	33.39	1.33	33.17	1.06	33.49	1.21
Eye surface	Mean	34.12	0.90	33.95	1.07	33.76	0.83	33.96	0.99
	SD	0.60	0.36	0.54	0.27	0.54	0.22	0.49	0.26
	Min	32.85	1.55	32.61	1.41	32.49	1.26	32.76	1.32
	Max	35.42	0.64	35.19	0.66	34.95	0.66	35.06	0.68
	Median'	**34.07**	**0.96**	**33.89**	**1.11**	**33.74**	0.83	**33.92**	**1.00**
	Mode	33.87	1.29	33.77	1.45	33.65	0.83	33.73	1.19
Orbital cavity	Mean	34.27	0.64	33.99	0.70	33.77	0.72	33.95	0.79
	SD	0.87	0.32	0.90	0.23	0.88	0.18	0.87	0.20
	Min	31.40	1.54	30.49	1.20	30.33	1.65	30.81	1.26
	Max	35.82	0.46	35.50	0.55	35.23	0.61	35.42	0.54
	Median^1^	**34.34**	**0.65**	**34.07**	**0.76**	33.84^*^	0.71	**34.03**	**0.81**
	Mode	34.68	0.98	34.69	1.09	34.38	1.13	34.53	1.04

* significant difference (p≤0.05) compared with one day before surgery,

1 the median temperature was selected for further analysis, Mean= mean temperature; SD= standard deviation; Min= minimum temperature; Max= maximum temperature; Median= median temperature, the middle value in a series is arranged from the lowest to the highest separating the same number of observations on both sides; Mode= the value that appears most often or the value that is most likely to be sampled

The area limited by two and five isotherms were analyzed to calculate the mean ratio of the area limited by the highest isotherm to the area limited by the lowest isotherm in the examined area of the eye. However, no significant differences were found between ratios obtained one day prior to and post-surgery (Table 2).

**Table 2:** Mean values of the area ratios for two and five isotherms at individual time points (highest to the lowest isotherm area) preand post-cataract surgery (n=36)

Surgery days/ Variable	Day -1		Day 1		Day 14		Day 28	
			Mean	SD	Mean	SD	Mean	SD
	Mean	SD						
Two isotherms	1.34	0.52	1.17	0.38	1.12	0.45	1.25	0.67
Five isotherms	1.57	0.94	1.21	0.64	1.08	0.51	1.23	0.77

Mean= mean temperature; SD= standard deviation.

**Figure 2:**
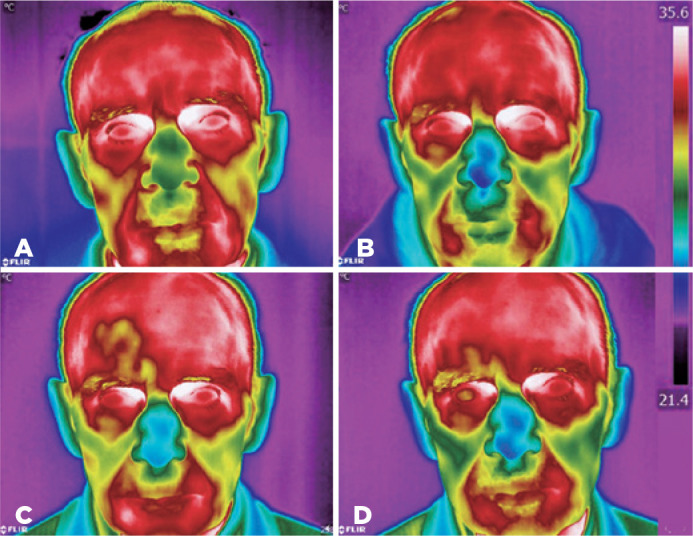
Thermographic image of a patient’s face on days -1 (A), 1 (B), 14 (C), and 28 (D) after cataract surgery.

Coefficients of correlation between the median surface temperature of examined areas and patients’ age at individual time points were determined. A weak negative correlation was found between the temperature of the cornea one day before surgery and the patient’s age. There was a moderate, but significant, correlation between the temperature of the eye and orbital cavity and patients’ age at 28 days after surgery. The correlation between surface temperature of the cornea and pa tient’s age remained unaltered prior to and one month after surgery (Table 3). Thermographic images of a patient’s face before and after the cataract surgery of the right eye are shown in figure 2.

**Table 3:** Correlations between the median surface temperature and patients’ age

Day -1			Day 1			Day 14			Day 28		
**Center of the cornea**	**Eye surface**	**Orbital cavity**	**Center of the cornea**	**Eye surface**	**Orbital cavity**	**Center of the cornea**	**Eye surface**	**Orbital cavity**	**Center of the cornea**	**Eye surface**	**Orbital cavity**
**-0.29s**	**-0.25**	**-0.18**	**-0.07s**	**-0.09s**	**-0.01**	**-0.03**	**-0.05s**	**0.06**	**-0.29**	-0.38^*^	-0.38^*^

s Spearman’s rank correlation coefficient for non-normally distributed data,

* correlation is significantly different (p≤0.05).

## DISCUSSION

The significant finding in our study was the reduction in median surface temperature of the orbital cavity 14 days after cataract surgery as compared with the preoperative temperature followed by an increase, on day 28 after surgery, to a temperature that was comparable to that measured before surgery, which indicated instability of the tear film, as previously reported.

There is a dearth in the literature reporting the impact of cataract surgery on ocular surface temperature, and available studies use ophthalmic techniques and tools that are no longer used. In 1994, Fujishima et al., demonstrated an increase in corneal surface temperature after extracapsular cataract extraction^(2)^ and more recently, Bissen-Miyajima et al. and Donnenfeld et al. reported on corneal and scleral burns caused by increased ocular surface temperature in the area around the phacoemulsification tip^(3)^, which the authors measured *in vivo* using the ocular temperature gradient during cataract surgery^(4,5)^. These methods of thermographic ana lysis determine only the temperature distribution on the ocular surface, but not inside the eye, where the temperature may be higher. Analyses of normal human eyes have demonstrated the highest temperature in the paranasal fields of the eye, and about 0.45°C-1.00°C higher temperature in the corneal limbus compared with the center of the cornea^(14,15)^. However, because the anterior chamber is smaller than the whole eye and the cornea is not vascularized, it can be assumed that the temperature inside the eye may not be significantly different from that measured in the anterior chamber projected on the ocular surface. To validate this hypothesis, using thermographic analysis and comparison of three different surgical procedures based on phacoemulsification, Corvi et al. compared the maximum temperatures measured in the anterior and posterior chambers using a thermal imaging camera and intraocular thermocouples during cataract surgery in pigs and found that while the temperature in the posterior chamber did not change during the procedure, regardless of the procedure, the temperature in the anterior chamber was comparable^(6)^. Their study revealed that the use of the Sovereign White Star phacoemulsification system with a bimanual technique was associated with the lowest temperature peaks and the lowest index of transmitted heat. The highest corneal temperature measured during phacoemulsification for this system was 44.9°C. Thermography was found to be a useful instrument that can be routinely used in the operating room for monitoring ocular surface temperature^(6)^.

In another study using thermography, Giannaccare et al., reported a significant change in postoperative ocular temperature in 26 patients, seven and 28 days after cataract surgery. Temperature was negatively correlated with the OSDI and directly related to TBUT^(7)^. While temperature immediately after and 10 s after eye opening was lower in the center of the cornea and nasal limbus, it was higher in the temporal limbus on days 7 and 28 after surgery. It was speculated that cooling in the central cornea could be due to tear film instability while higher temperature in the temporal limbus may be attributed to tissue inflammation in response to surgery^(7)^. These findings were also corroborated by Shih et al. who demonstrated a significant correlation between the decrease in ocular surface temperature 10 s after eye opening and the instability of the tear film. Temperature in the temporal region of the eye increased in the first week after surgery and returned to normal one month after surgery. The authors hypothesized that temperature increase was associated with postsurgical inflammation and increased vascular and metabolic acti vity around the surgical incision in the cornea^(8)^.

Sniegowski et al., found no significant differences in ocular surface temperature between healthy phakic and pseudophakic patients one month after cataract surgery, indicating normalization in temperature. The mean ocular surface temperature was in the range of 32.9°C-36.0°C in all groups in this study, which is in line with previous observations (34.02°C ± 0.22°C)^(9)^. However, there was a weak negative correlation between age and ocular surface temperature in our study, while previous reports did not find any significant difference in gender, ethnicity and between the right and left eyes^(10,11)^.

No correlation was reported between the thickness and density of the cornea and the length of the anterior chamber of the eye and the ocular surface temperature^(12)^. Environmental conditions are also important, since room temperature may have a positive correlation with ocular surface temperature of up to 0.15°C-0.20°C for each Celsius degree rise. Air currents and humidity in the room may disturb local heat transfer; therefore, adaptation of patients is recommended before measurements are taken with the thermographic camera^(13)^.

Our study revealed an age-related reduction in corneal temperature though the correlation between these two factors was not significant, possibly due to the small sample size. An age-related decrease in ocular surface temperature by 0.01°C-0.023°C per year, that was more pronounced in middle-aged and elderly subjects^(16)^, was attributed to increasing instability and evaporation of the tear film^(17-19)^.

The incidence of dry eye syndrome increases rapidly after cataract surgery. Thermal imaging revealed that dry eyes or eyes with pathologies have a lower temperature than that of normal eyes, which may be explained by the lower emissivity of the unstable tear film^(20,21)^. In this study, the lacrimal river line narrowed and the BUT and STI decreased in the patients examined. Impression cytology suggests the presence of serious squamous me taplasia in the epithelial layer of the eye, especially in the lower region of the cornea^(22)^, which corroborates rep orts of abnormalities on the corneal surface after phacoemulsification^(23)^. The authors of the study reported significantly detrimental changes in all parameters of corneal sensitivity and tear physiology 3 days after phacoemulsification. Whereas the physiological parameters of tear film returned to normal within one month, corneal sensitivity improved over subsequent weeks, but did not return to normal within 3 months of follow-up^(23)^.

Increased thermal emissions have also been reported in ocular inflammation^(24,25)^. A transient inflammation after cataract surgery produces a significant amount of pro-inflammatory cytokines, such as IL-1β, IL-6, and PGE2, in the aqueous humor^(26-28)^, which may explain the higher temperature detected using a thermal imaging camera in the temporal quadrant around the corneal incision site comprising complex vasculature^(7,8)^; however, we were unable to confirm these observations.

In this study, we were able to show that a thermal imaging camera can be a helpful tool in monitoring temperature during cataract surgery, as well as in assessing the condition of the ocular surface after cataract phacoemulsification with simultaneous microsurgical insertion of a foldable intraocular lens.

The reduction in ocular surface temperature toward the end of post-cataract surgery follow-up may be associated with increased instability of the tear film after phacoemulsification. Therefore, patient awareness regarding the possibility of clinical symptoms of dry eye syndrome during the first month after surgery should be part of clinical management of cataract surgery. Ocular surface temperature did not increase after cataract surgery, suggesting an absence of significant inflammation, and the temperature about one month after cataract surgery was comparable to the temperature before surgery. Nevertheless, the negative correlation between age and ocular surface temperature should be of concern in the elderly.
